# Prognostic Model and Immune Infiltration of Ferroptosis Subcluster-Related Modular Genes in Gastric Cancer

**DOI:** 10.1155/2022/5813522

**Published:** 2022-10-13

**Authors:** Huachu Deng, Yongjian Lin, Fu Gan, Baibei Li, Zhenshan Mou, Xingan Qin, Xiaoyu He, Yunchao Meng

**Affiliations:** ^1^Department of Gastrointestinal and Gland Surgery, The First Affiliated Hospital of Guangxi Medical University, Nanning 530021, China; ^2^Department of Urology Surgery, The Affiliated Hospital of Youjiang Medical University for Nationalities, Baise 533000, China; ^3^Department of Hepatobiliary, The First Affiliated Hospital of Guangxi Medical University, Nanning 530021, China; ^4^Department of Thoracic Surgery, The Shenzhen Bao'an District Songgang People's Hospital, Shenzhen 518105, China; ^5^Department of Gastroenterology, Nanxishan Hospital of Guangxi Zhuang Autonomous Region, Guilin 541002, China

## Abstract

**Background:**

Gastric cancer (GC) is one of the gastrointestinal tumors with the highest mortality rate. The number of GC patients is still high. As a way of iron-dependent programmed cell death, ferroptosis activates lipid peroxidation and accumulates large reactive oxygen species. The role of ferroptosis in GC prognosis was underrepresented. The objective was to investigate the role of ferroptosis-related genes (FRGs) in the prognosis and development of GC.

**Methods:**

Datasets of GC patients were obtained from the Gene Expression Omnibus (GEO) and The Cancer Genome Atlas (TCGA) database that include clinical information and RNA seq data. Through nonnegative matrix factorization (NMF) clustering, we identified and unsupervised cluster analysis of the expression matrix of FRGs. And we constructed the co-expression network between genes and clinical characteristics by consensus weighted gene co-expression network analysis (WGCNA). The prognostic model was constructed by univariate and multivariate regression analysis. The potential mechanisms of development and prognosis in GC were explored by Kyoto Encyclopedia of Genes and Genomes (KEGG) analysis, gene ontology (GO), tumor immune microenvironment (TIME), and tumor mutation burden (TMB).

**Results:**

Two molecular subclusters with different expression patterns of FRGs were identified, which have significantly different survival states. Ferroptosis subcluster-related modular genes were identified by WGCNA. Based on 8 ferroptosis subcluster-related modular genes (collagen triple helix repeat containing 1 (CTHRC1), podoplanin (PDPN), procollagen-lysine,2-oxoglutarate 5-dioxygenase 2 (PLOD2), glutamine-fructose-6-phosphate transaminase 2 (GFPT2), ATP-binding cassette subfamily A member 1 (ABCA1), G protein-coupled receptor 176 (GPR176), serpin family E member 1 (SERPINE1), dual specificity phosphatase 1 (DUSP1)) and clinicopathological features, a nomogram was constructed and validated for their predictive efficiency on GC prognosis. Through receiver operating characteristic (ROC) analysis, the results showed that the area under the curve (AUC) of 1-, 3-, and 5-year survival were 0.721, 0.747, and 0.803, respectively, indicating that the risk-scoring model we constructed had good prognosis efficacy in GC. The degree of immune infiltration in high-risk group was largely higher than low-risk group. It indicated that the immune cells have a good response in high-risk group of GC. The TMB of high-risk group was higher, which could generate more mutations and was more conducive to the body's resistance to the development of cancer.

**Conclusion:**

The risk-scoring model based on 8 ferroptosis subcluster-related modular genes has shown outstanding advantages in predicting patient prognosis. The interaction of ferroptosis in GC development may provide new insights into exploring molecular mechanisms and targeted therapies for GC patients.

## 1. Introduction

GC is one of the gastrointestinal tumors with the highest mortality rate [1]. The number of GC patients is still high. By 2022, about 26,380, new cases are expected with a mortality rate of 42% [2]. Although the current predominant treatment for GC takes the standard surgical strategy combined limited lymph node dissection strategy [3], there are still patients with heterogeneous prognosis, such as distant metastases, significant drug resistance, and toxic effects [4]. Meanwhile, early GC commonly lacked dysphagia, weight loss, and typical gastrointestinal symptoms. So most people are diagnosed with advanced GC. The survival for advanced GC is always low [5]. Therefore, identifying high-tech diagnostic and prognostic biomarkers is crucial for the management of GC. Relevant advances in the field of tumor mutational burden and gene mutation might be immediately applicable to guide immunotherapeutic effect of GC [6, 7].

Programmed cell death (PCD) includes cell apoptosis, necroptosis, autophagy, pyroptosis, cuproptosis, and ferroptosis. They have different morphologies and biochemical characteristics. For example, apoptosis is usually associated with cell contraction, while necrotizing apoptosis involves cell swelling and leakage of cell contents [8]. Necroptosis is a form of regulated cell death that critically depends on receptor-interacting serine-threonine kinase 3 (RIPK3) and mixed lineage kinase domain-like (MLKL) and generally manifests with morphological features of necrosis [9]. Autophagy is a process by which cellular material is degraded by lysosomes or vacuoles and recycled [10]. Cuproptosis is a new type of cell death and is characterized by the dependence on mitochondrial respiration and protein lipoylation [11]. As a way of iron-dependent programmed cell death, ferroptosis activates lipid peroxidation and accumulates large reactive oxygen species [12]. The main mechanism of ferroptosis is to catalyze the production of lipid peroxidation of highly expressed unsaturated fatty acids on cell membranes under the action of divalent ferroxygenase or ester oxygenase, thereby inducing cell death. In addition, it also showed a decrease of GPX4, the core enzyme regulating the antioxidant system (glutathione system) [13]. The main characteristics of ferroptosis were as follows: (1) In terms of cell morphology, ferroptosis could lead to smaller mitochondria, increase membrane density, and reduce crest. There was no obvious morphological change in the nucleus. (2) In terms of cell components, iron death showed increased lipid peroxidation and ROS. This process is present in tumor development and therapeutic response, including genetic mutations, stress response pathways, and epithelial-to-mesenchymal transition [14]. Ferroptosis was closely associated with antitumor, drug resistance and metastasis. Ni et al. have illustrated miR-375/SLC7A11 regulatory axes triggering gastric cancer stem cell iron sagging, attenuated metastasis, and drug resistance [15]. Other studies have shown that poor prognosis in patients with GC was largely associated with cancer cell antiferroptosis, and its underlying mechanisms may involve alterations in cancer stem cells and regulation of cell cycle-related proteins [16]. In addition, inducing ferroptosis was one of the main mechanisms mediating antitumor activity. Liu et al. found that Jiyuan oridonin A (JDA) was a natural compound isolated from Jiyuan Rabdosia rubescens with antitumor activity, which could inhibit the growth of GC cells by inducing ferroptosis [17]. However, the role of ferroptosis in GC prognosis was underrepresented, especially with the presence of mutant types of GC. Therefore, in this study, a novel GC prognosis model was constructed through ferroptosis subgroup-related module genes.

In this study, we aimed to identify FRG co-expression modules in GC through WGCNA, develop a risk-scoring model to quantify the level of ferroptosis in individual patients, and explore its prognostic role in GC patients. In addition, functional studies were conducted on tumor immune microenvironment and mutation burden to initially elucidate the mechanisms that affect prognosis, providing a basis for clinical diagnosis, personalized immune targeted therapy, and antitumor drug resistance.

## 2. Methods

### 2.1. Sources of Data

The mRNA sequencing and somatic mutation data of GC patients were obtained from the Cancer Genome Atlas (TCGA) (https://tcga-data.nci.nih.gov/tcga/), which contains 375 GC samples. And these samples were used as a training set for this study, while the validation set was selected from the GSE84437 dataset in the Gene Expression Omnibus (GEO) database (https://www.ncbi.nlm.nih.gov/geo/). The dataset contains gene expression data from 433 patients with GC. 383 FRGs were derived from ferroptosis database (http://www.zhounan.org/ferrdb/) [18–20]. The “limma” package was used for integration and difference analysis between datasets [21].

### 2.2. Identification of Subclusters of FRGs in GC

The NMF algorithm has a great advantage in the distance [22]. The “NMF” package in R was used to identify and unsupervised cluster analysis of the expression matrix of FRGs obtained from the TCGA dataset. Each parameter was selected in the following way, using the “brunet” package in R, with the number of iterations (nrun) set to 10 and ranks set from 2 to 10. Then the new subcluster classification was obtained.

### 2.3. Identification of Ferroptosis Subcluster-Related Modular Genes in GC

The differential expression genes with different ferroptosis genotyping between cluster 1 (C1) and cluster 2 (C2) group were identified with the “limma” R package according to the cut-off value FDR<0.05, log2|fold change (FC)| ≥2. The co-expression network between genes and clinical characteristics was constructed using the “WGCNA” R package [23], and the samples with an expression less than 0.5 were removed. Subsequently, we computed the topological matrix of the scale-free distribution. We used the “PickSoftThreshold” function to select the optimal soft threshold *β* and then calculated the Pearson correlation coefficient for each gene. We construct a neighbor-joining matrix using weighted correlation coefficients. Then, a topological overlap matrix was constructed based on the neighbor-joining matrix to construct a clustering tree. The number of genes in the module greater than 150 was retained, and the modules with similarities greater than 0.25 were merged. Finally, the significant models were identified, and the genes of the significant modules were extracted.

The “VennDiagram” package was applied to calculate the intersection genes of difference genes between C1 and C2 and the FRGs of the significant modules. That is the ferroptosis subcluster-related modular genes.

### 2.4. Construction and Validation of a Prognostic Model

The TCGA dataset was used as the training set, and the genes associated with patients' prognosis were screened out through COX regression analysis. Using the least absolute shrinkage and selection operator (LASSO) regression method, these genes were analyzed by the “glmnet” software package in R. And the *λ* min with the lowest error was chosen after 10-fold cross-validation [24] to construct a stable prognostic model. Followed by multivariable cox regression analysis to construct the best risk-scoring model and calculate regression coefficient for each gene regression coefficient. The risk score in the model was calculated by the following formula: riskScore = ∑_*x*=1_^*n*^(coef mRNA × Expr mRNA). The risk score for each sample was calculated using this formula, and the low- and high-risk groups were divided by the median [25]. Through “survival” and “timeROC” package in R, the Kaplan-Meir (K-M) survival curve and ROC curve were, respectively, plotted to determine the efficacy of the model. In addition, for more intuitive prediction, we incorporated clinical characteristics (including age, gender, tumor location, and metastasis) into the model, constructed a nomogram using the “Regplot” package in R, and validated the stability with calibration and ROC curves.

To demonstrate accuracy of the model, we used the GSE84437 dataset for external validation of the risk model construction, including survival curves and ROC curves.

### 2.5. Functional Analysis of Prognostic FRGs

Functional enrichment analysis of GO and KEGG pathways were performed to describe functions of prognostic FRGs. Through the “clusterProfiler” package in R, functional enrichment analysis of prognostic FRGs was performed. Items with adjusted *P* < 0.05 were selected from the enrichment results for display.

### 2.6. Analysis between Immune Infiltration and Risk Score of GC

Through the single-sample GSEA (ssGSEA) algorithm, the “GSVA” package of R was applied to calculate the relative content of different kinds of immune cells in the TCGA cohorts and explore the differences in immune infiltration. And we applied the “estimate” package to perform ESTIMATE algorithm to analyze the data of TCGA cohorts and evaluate the TIME from three aspects: tumor purity, immunity score, and matrix score.

### 2.7. Tumor Mutational Burden (TMB) Analysis of GC

Based on the corresponding mutation data, we calculated their nonsynonymous mutations to determine the TMB of GC patients with different ferroptosis subclusters. We extracted GC patient driver gene data from the R “maftool” package and compared the somatic changes in driver genes of different ferroptosis subtypes. Finally, the overall mutation level was represented by the top 20 driver genes by mutation frequency.

### 2.8. Statistical Analysis

All data were statistically analyzed using R (Version 3.6.2). K-M method, Log-rank test, and Cox regression were used to analyze the prognosis of each characteristic value, the survival curve, and the independent prognostic factors, respectively. ROC curve analysis was used to predict overall survival with R package “pROC”. Continuous variables (e.g., age, gender, stage, and tumor grade) were transformed into dichotomous variables. Student's *t*-test and chi-square test were adopted to compare differences in pathology and molecular characteristics between different patient groups. And Welch's t test was used when appropriate. When *P* < 0.05, analysis was considered statistically significant.

## 3. Results

### 3.1. Construction of Subclusters of FRGs in GC

**Figure 1:**
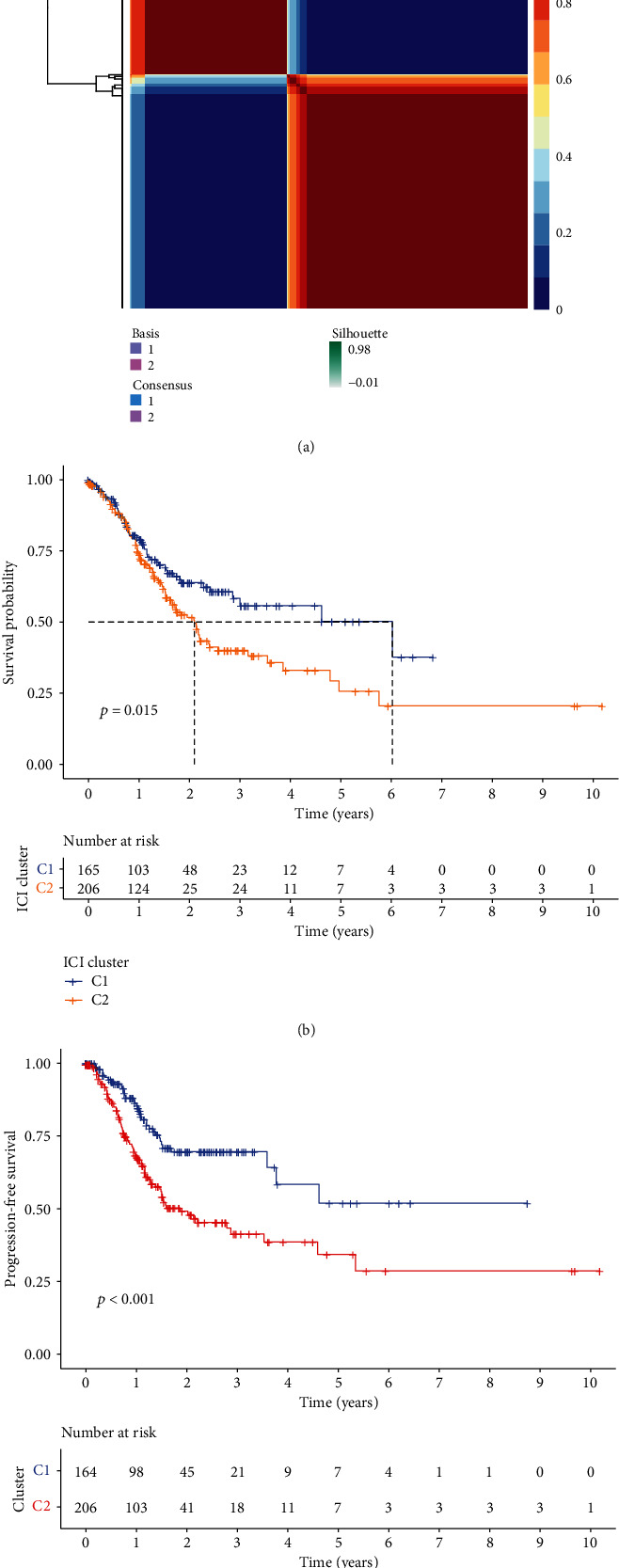
Identification of ferroptosis-associated subclusters. (a) Through NMF consensus clustering using FRGs, the optimal k value was determined to be 2. Patients were divided into C1 and C2. (b) K-M survival curves of OS in C1 and C2. (c) K-M survival curves of PFS in C1 and C2. C1: cluster 1; C2: cluster 2; OS: overall survival; PFS: progression-free survival; GC: gastric cancer; NMF: nonnegative matrix factorization; K-M: Kaplan-Meir; FRG: ferroptosis-related genes.

Through NMF clustering analysis of FRGs, 375 GC samples from TCGA were divided into two subclusters: C1 and C2 (Figure 1(a)). Survival analysis showed that there were significant differences between the C1 and C2 in the overall survival (OS) and progression-free survival (PFS). The survival rate of the C1 is superior to the C2, and K-M curve is shown in Figures 1(b) and 1(c).

### 3.2. Difference Analysis of Subclusters in GC and Construction of Ferroptosis-Related Modules by WGCNA

**Figure 2:**
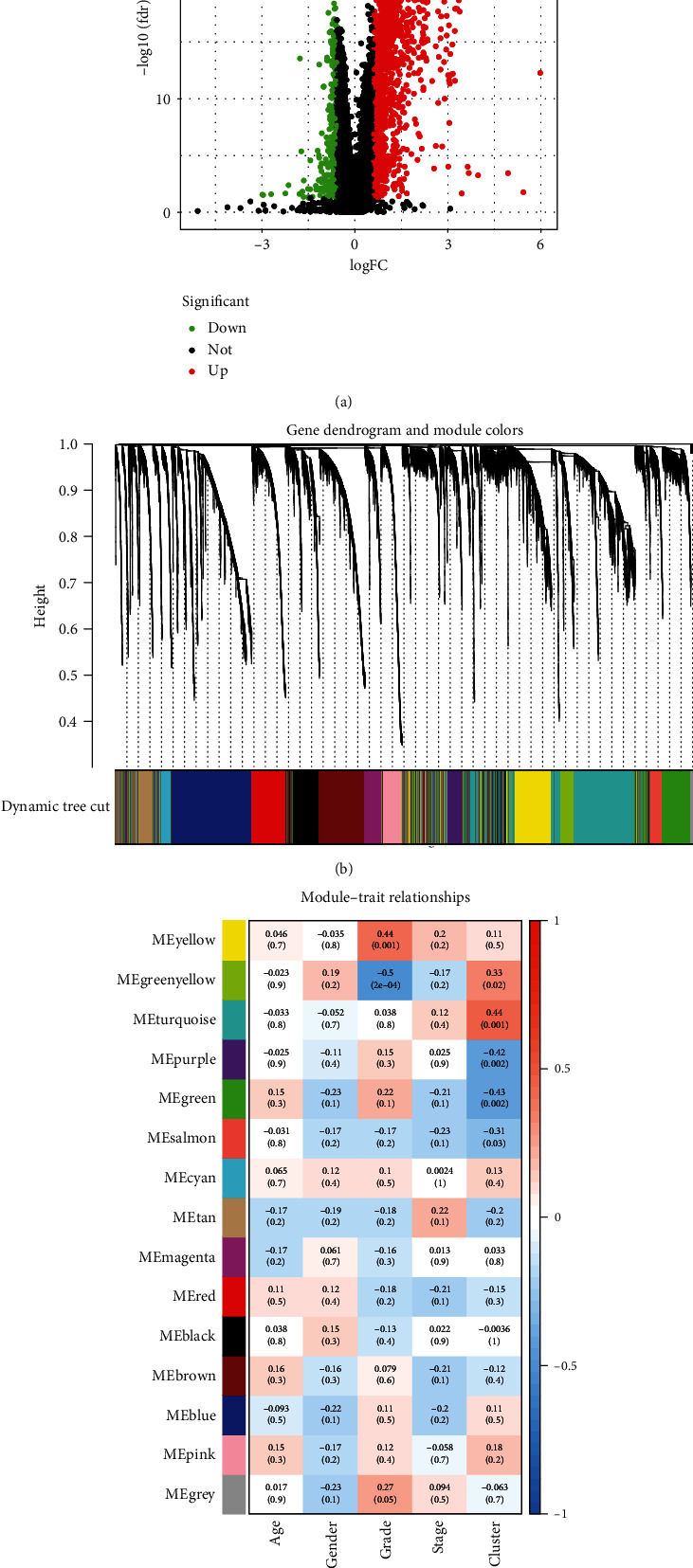
Identification of differential molecular subcluster genes associated with ferroptosis. (a) Volcano plots of mRNA-seq differential analysis for C1 and C2. (b) Based on the hierarchical clustering analysis of the TCGA dataset, genes with similar characteristics are assigned to modules of the same color. (c) Heat map of correlations between eigenvalues and individual modules. C1: cluster 1; C2: cluster 2; TCGA: the Cancer Genome Atlas.

Difference analysis of C1 and C2 was performed through the “limma” package, and a total of 1846 differential genes met the requirements, and the differential genes were displayed in a volcanic map (Figure 2(a)). During the analysis of WGCNA, we calculated the soft threshold *β* = 5 using *R*, and then obtained a hierarchical cluster tree by dynamic cutting method (Figure 2(b)), and combined similar modules to obtain a total of 15 modules. From the Pearson correlation analysis matrix of the module features, the green yellow and yellow modules were related to the ferroptosis-related phenotype in GC. The green yellow module showed the highest correlation (*R* = −0.5, *P* < 0.001) (Figure 2(c)). To construct a risk-scoring model associated with ferroptosis, 555 genes in the green yellow module were taken to intersect with 1846 DEGs for C1 and C2, and finally, 80 key genes were obtained.

### 3.3. Construction and Validation of Risk-Scoring Model

**Figure 3:**
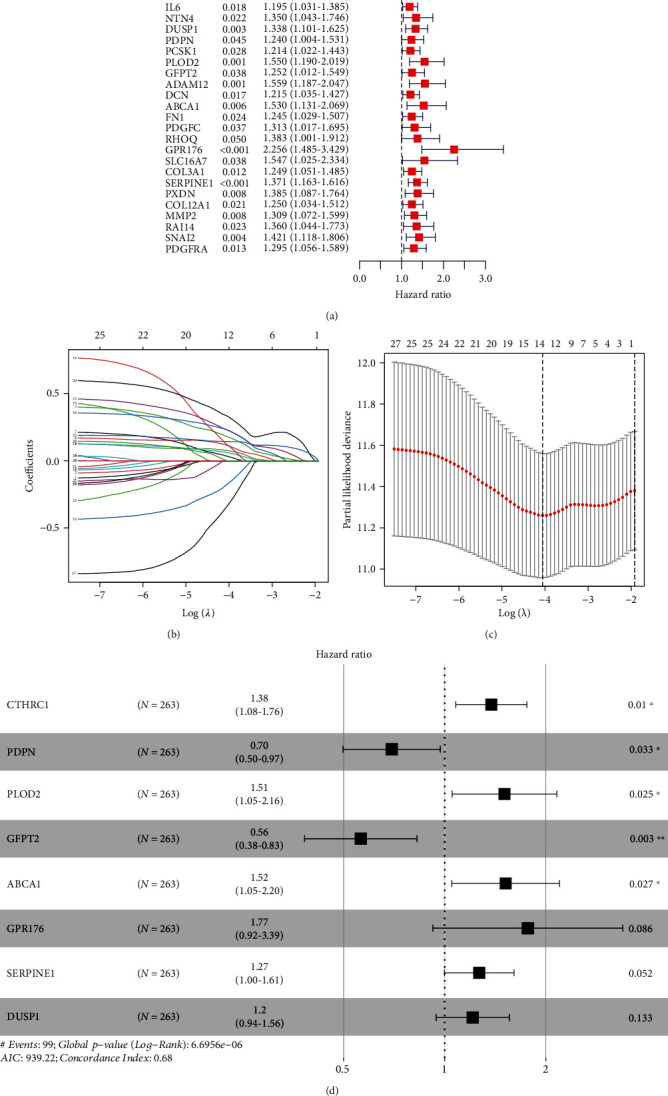
Screening of prognostic FRGs. (a) Univariate Cox regression analysis screened out 29 prognostic FRGs. (b) Trajectory changes of 8 genes. (c) Confidence interval for each *λ* value. (d) Multivariate Cox regression analysis screened out 8 prognostic FRGs. C1: cluster 1; C2: cluster 2; FRGs: ferroptosis-related genes.

**Figure 4:**
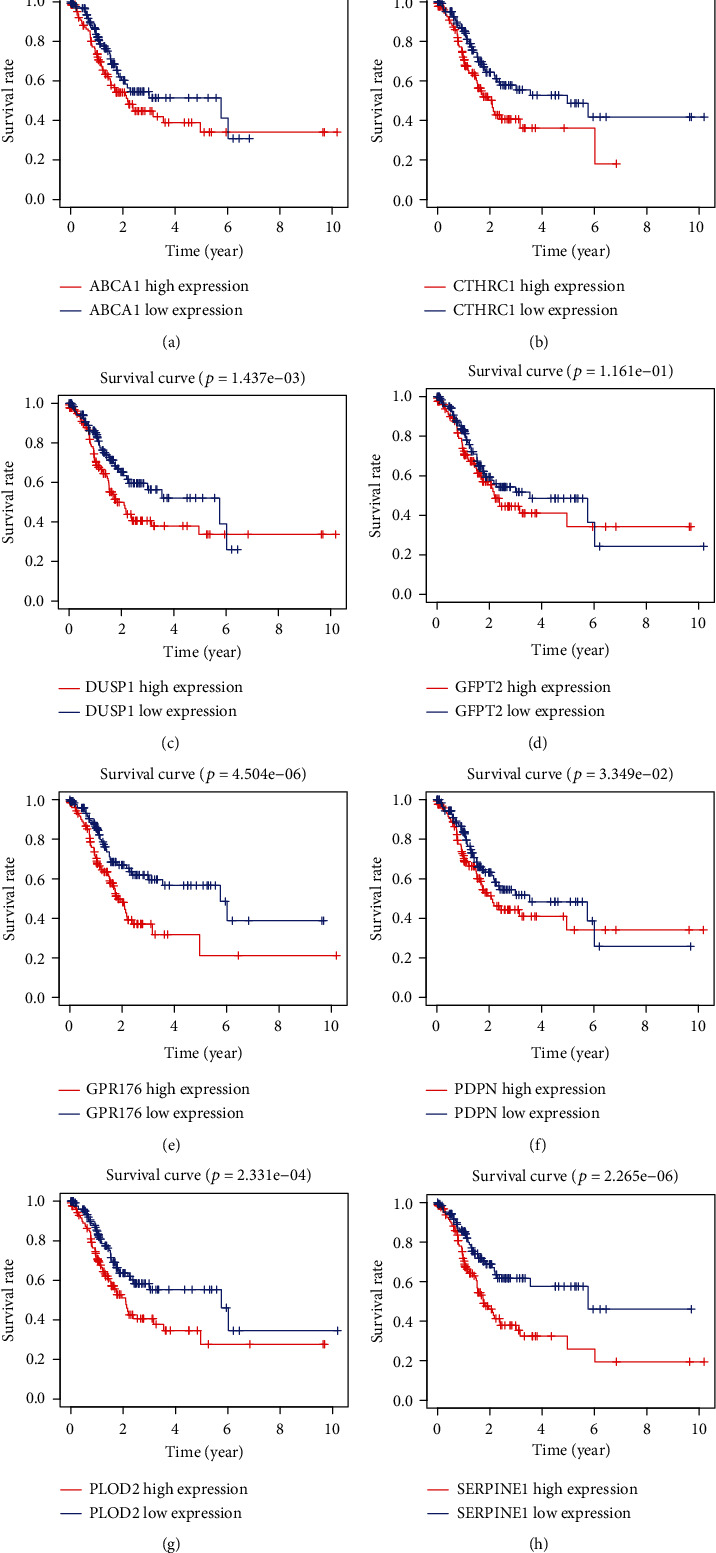
K-M survival curves of 8 genes with independent prognostic potency. K-M: Kaplan-Meir.

**Figure 5:**
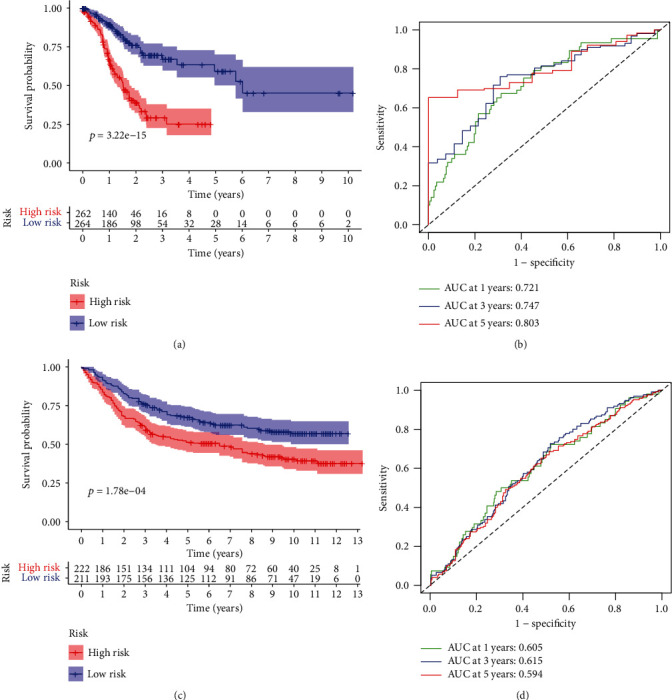
Identification and validation of FRGs signatures. (a) K-M survival curves of low- and high-risk groups in TCGA total cohort. (b) ROC curves of 1-, 3-, and 5-year survival for TCGA total cohort. (c) K-M survival curves of low- and high-risk groups in GEO cohort. (d) ROC curves of 1-, 3-, and 5-year survival for GEO cohort. FRGs: ferroptosis-related genes; K-M: Kaplan-Meir; TCGA: the Cancer Genome Atlas; GEO: Gene Expression Omnibus; ROC: receiver operating characteristic.

Through univariate Cox regression analysis, with *P* < 0.01 as a filter, 29 genes associated with GC prognosis were obtained in the training set (Figure 3(a)). To prevent the model from overfitting, we took Lasso regression analysis to test these 29 genes and determined that there was no over-fitting of the model for these 29 genes (Figures 3(b) and 3(c)). Finally, we identified 8 prognostic-related genes using multivariate Cox regression analysis (Figure 3(d)) and plotted K-M curves for 8 genes (Figure 4). Based on these 8 genes, we constructed a risk-scoring model for GC. Risk Score = 0.320^∗^CTHRC1 + (−0.364)^∗^PDPN + 0.410^∗^PLOD2 + (−0.575)^∗^GFPT2 + 0.418^∗^ABCA1 + 0.570^∗^GPR176 + 0.237^∗^SERPINE1 + 0.192^∗^DUSP1. According to the median risk score value, the GC samples were divided into high- and low-risk group. And we have drawn the K-M curve through “survival” package of R (Figure 5(a)). It was demonstrated that there was a significant survival difference between the low- and high-risk groups. In addition, the ROC analysis showed that the AUC of 1-, 3-, and 5-year survival were 0.721, 0.747, and 0.803, respectively. (Figure 5(b)) It meant that the model we constructed had great diagnostic efficacy in GC.

To ensure the accuracy of the risk-scoring model, we verified the model on an external validation set (GEO database) and found that the survival of low- and high-risk groups of the GEO dataset was significantly different in the K-M survival curve (Figure 5(c)). The ROC analysis showed that the AUC of 1, 3, and 5 years were 0.605, 0.615, and 0.594, respectively (Figure 5(d)). It demonstrated that the prognostic model of FRGs had good accuracy in the validation set.

### 3.4. Nomogram Construction through Risk Score and Clinicopathological Features

**Figure 6:**
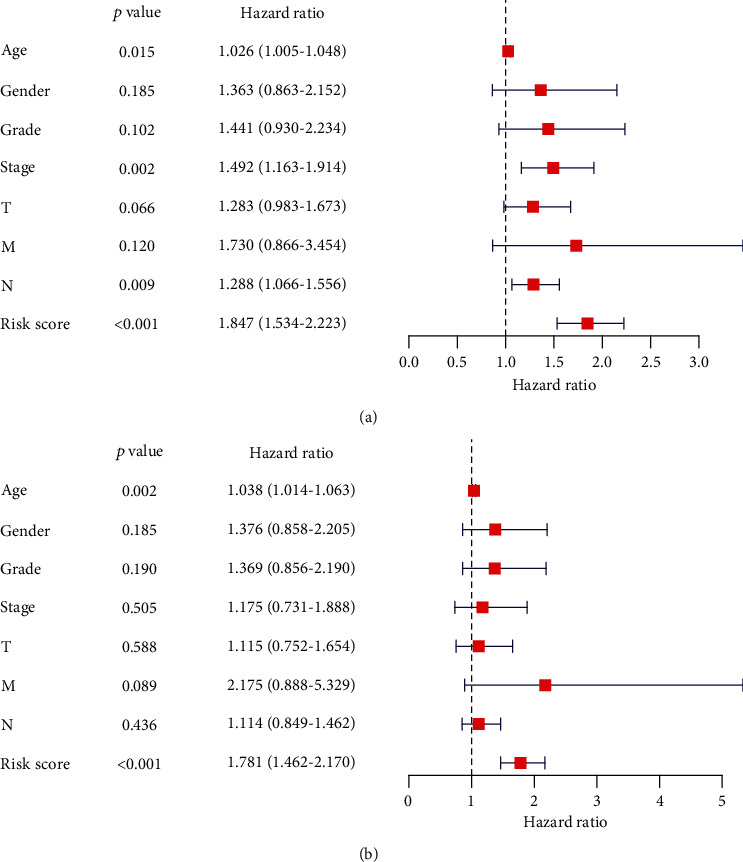
(a) Univariate and (b) multivariate Cox regression analysis of clinicopathological features.

**Figure 7:**
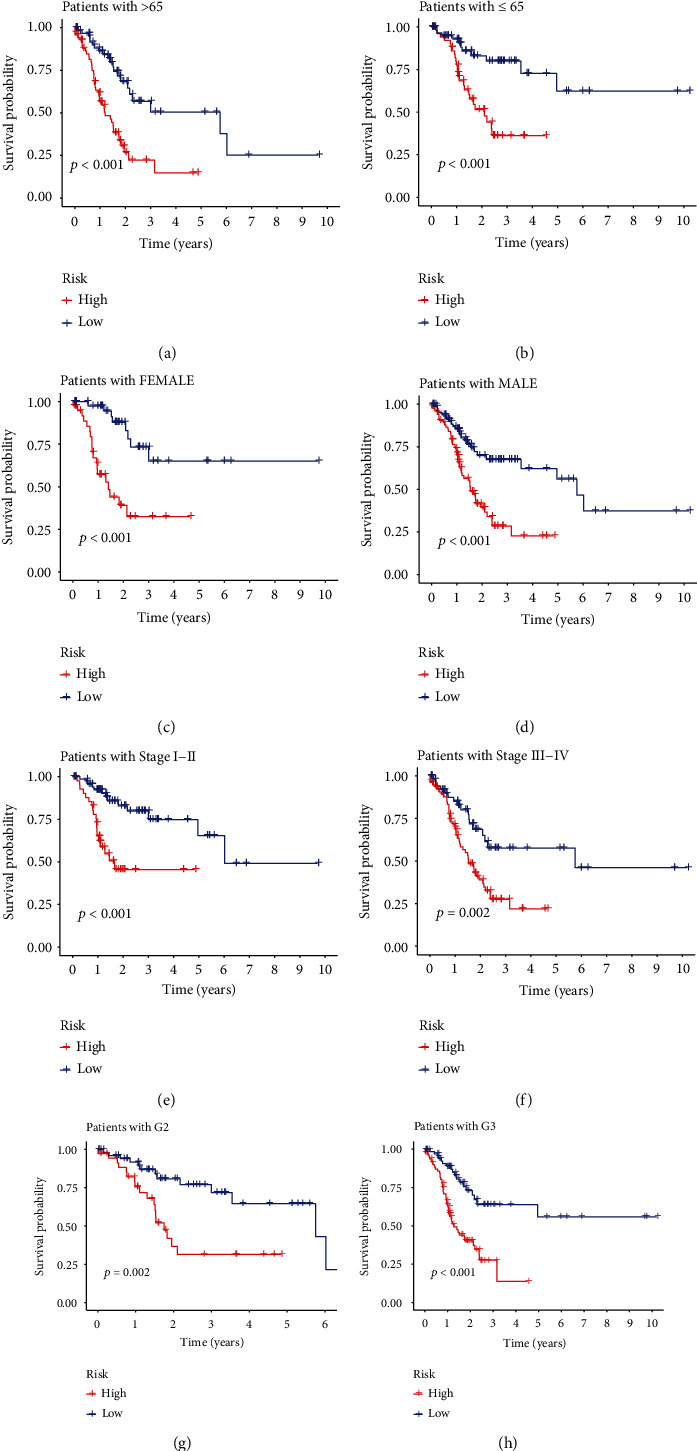
Survival analysis of clinicopathological features. (a–h) K-M survival curves of low- and high-risk groups at age (≤65, >65), gender (female, male), stage (stage I-II, stage III-IV), and grade (G2, G3).

**Figure 8:**
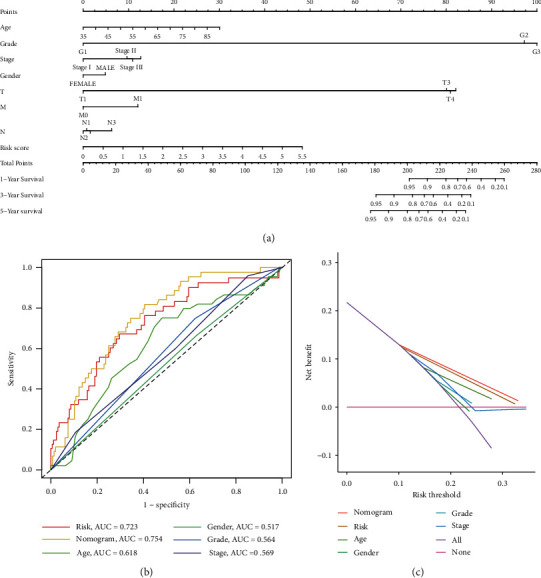
Construction and validation of a prognostic model for GC patients. (a) Nomogram for predicting GC patients for 1-, 3-, and 5-year survival based on the TCGA total cohort. (b) ROC curve containing age, gender, grade, stage, risk score, and nomogram. (c) DCA of the nomogram. The net benefit was plotted versus the threshold probability. GC: gastric cancer; TCGA: the Cancer Genome Atlas; ROC: receiver operating characteristic; DCA: decision curve analysis.

In the risk-scoring model constructed based on 8 ferroptosis subcluster-related modular genes, we incorporated clinicopathological features in GC. Then, the analysis of eigenvalues was followed by Cox regression analysis. Prognosis of GC was correlated with age and risk score. And both of them were independent prognostic factors (Figure 6). At the same time, the analysis of K-M survival curves showed significant differences in survival at different age, gender, tumor grade, and stage (Figure 7). Based on clinicopathological features and risk score, we established a nomogram that could predict the prognosis of GC (Figure 8(a)). The 1-, 3-, and 5-year survival of GC could be predicted by scoring each characteristic value. Besides, we used ROC curves to determine their accuracy (Figure 8(b)). Through results of decision curve analysis (DCA), we believe that the nomogram has high clinical application value (Figure 8(c)). In conclusion, the model could accurately predict the survival of GC patients.

### 3.5. Functional Enrichment Analysis

**Figure 9:**
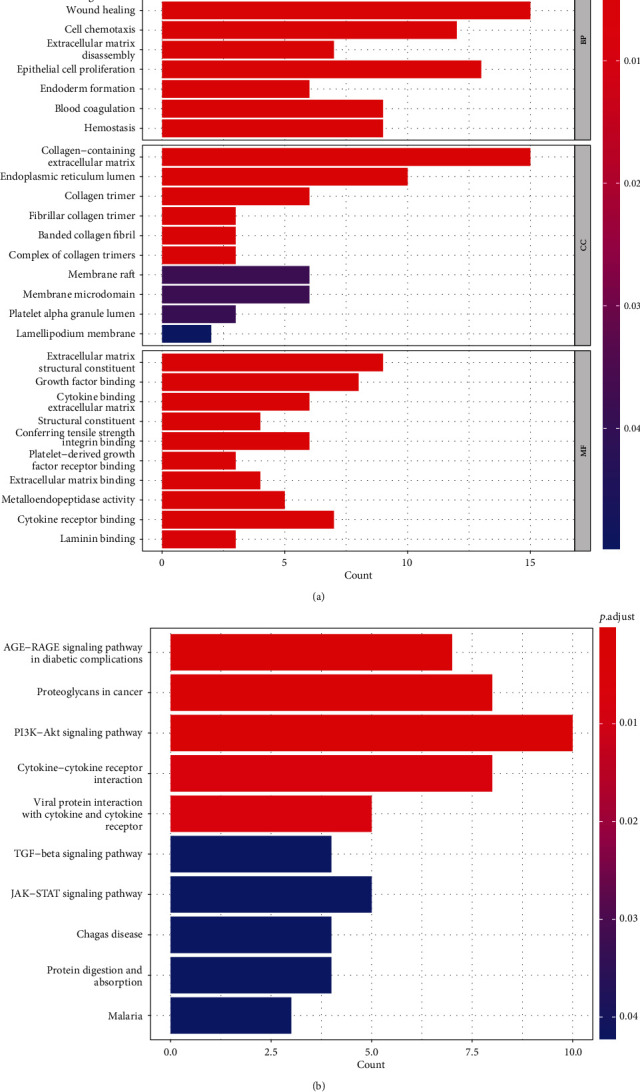
GO and KEGG analysis of prognostic FRGs. (a) Histogram of GO enrichment analysis for prognostic FRGs. (b) Histogram of KEGG enrichment analysis for prognostic FRGs. GO: gene ontology; KEGG, Kyoto Encyclopedia of Genes and Genomes; FRGs: ferroptosis-related genes.

We preformed GO and KEGG enrichment analysis to annotate the biological characteristics of 8 genes by “clusterProfiler” package in R (Figures 9(a) and 9(b)). GO enrichment analysis indicated that these genes were mainly enriched in extracellular matrix, organization extracellular structure, organization external encapsulating, wound healing, and other biological processes (BP). Besides, they were enriched in collagen-containing extracellular matrix, endoplasmic reticulum lumen, collagen trimer, and other cellular constituents (CC). Molecular functions (MF) were enriched in growth factor binding, extracellular matrix structural constituent, and cytokine binding extracellular matrix. KEGG analysis indicated that they were related to PI3K−Akt, TGF, JAK−STAT, and other metabolic signaling pathway.

### 3.6. Analysis of Immune Cell Infiltration

**Figure 10:**
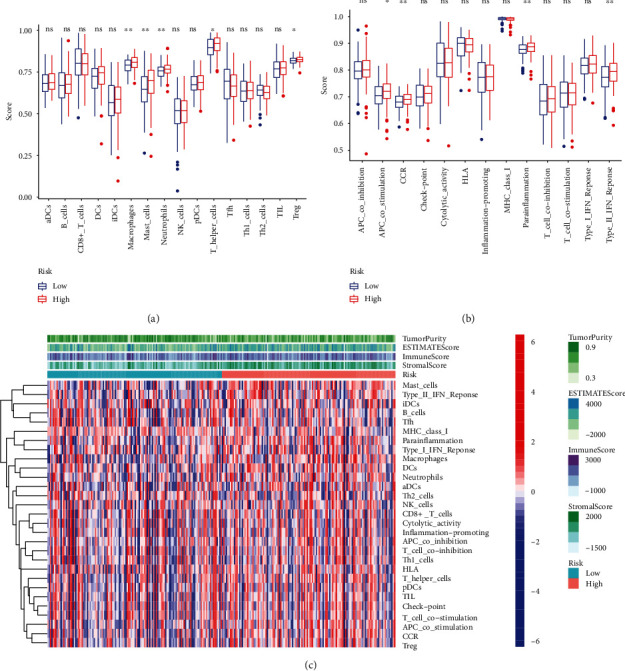
Analysis of immune infiltration. (a) Analyze the immune differences between low- and high-risk groups from 16 immune cells. (b) Analyze the immune differences between low- and high-risk groups from 13 immune-related functions. (c) Heat map of stromal score, immune score, and estimate score in low- and high-risk groups.

Through ssGSEA algorithm, we obtained 16 kinds of immune cells and 13 kinds of immune-related mechanisms in GC. It demonstrated that the degree of immune infiltration in low-risk group was lower than that in high-risk group (Figures 10(a) and 10(b)). Macrophages, mast cells, neutrophils, Treg, and T helper cells showed significantly different distribution, and T cells were the most abundant immune cells in GC tissue infiltration. The result suggested that the immune cells have a good response in high-risk group of GC. According to the ESTIMATE algorithm of “estimate” R package, the heat map showed that compared with the low-risk group, the high-risk group had lower tumor purity and higher stromal score, immune score, and estimate score. The tumor purity of TME decreased, and the infiltration of stromal and immune cells increased significantly (*P* < 0.05) (Figure 10(c)).

### 3.7. Analysis of TMB for GC

**Figure 11:**
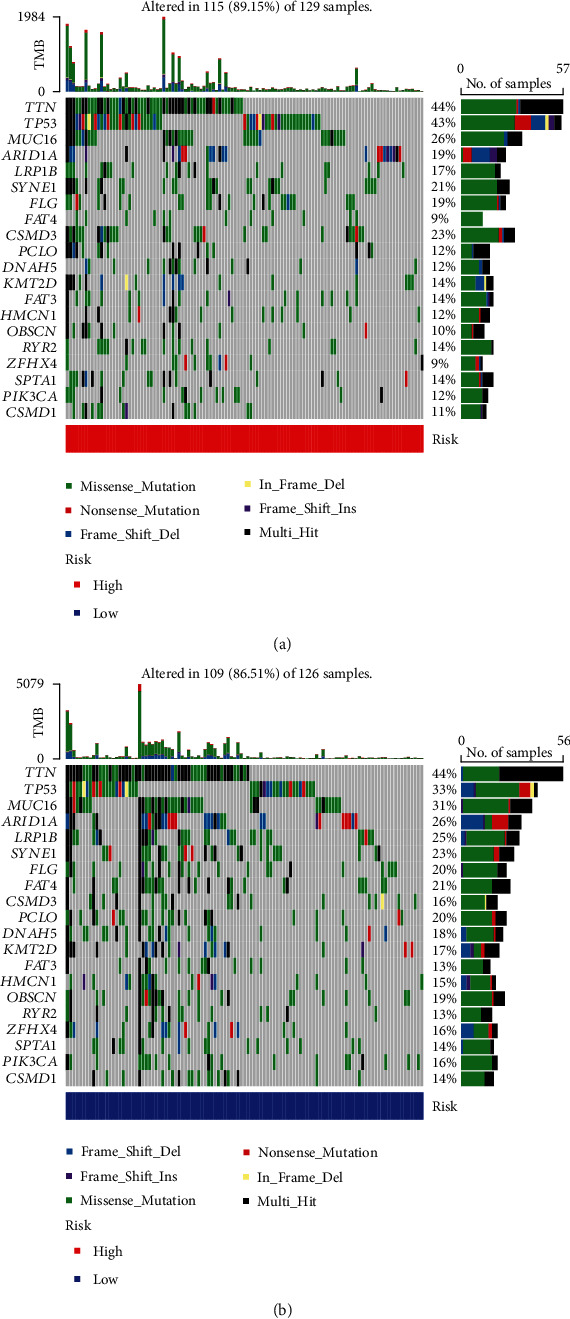
The waterfall diagram demonstrates the top 20 driver genes with the highest mutation frequency in high-risk group (a) and low-risk group (b).

The TMB was a way for somatic cells to increase the types of antigens by mutation and thus resist cancer [26]. The TMB was calculated and compared between two groups by “maftools” package (Figures 11(a) and 11(b)). Higher TMB could generate more mutations and was more conducive to the body's resistance to the development of cancer. In this study, the waterfall diagrams showed that TTN and TP53 genes in the two groups had the highest mutation rates. TTN gene was 44% of mutations in both groups, while TP53 was 43% of the mutations in high-risk group and 33% of the mutations in low-risk group.

## 4. Discussion

GC is a common tumor worldwide, with a large number of cases, especially in East Asian countries. The prognosis of GC varies widely between countries. Early detection and intervention could improve the prognosis [1]. In this study, we developed a unique prognostic model for FRG in GC using TCGA and GSE84437 cohort data. Then, we constructed a quantitative scoring system and further evaluated the effect of FRG on immune infiltration. Ferroptosis, a novel form of cell death, is characterized by unique morphology, gene expression, and molecular pathways. Previous studies identified that GSH, GPX4 activity inhibition, and iron-dependent ROS burst were the critical factors inducing ferroptosis [27]. FRGs are associated with TIME and TMB, which is helpful to predict the prognosis of GC. Mutations in DNA damage-responsive genes are the main cause of elevated TMB and can be used to predict immune checkpoint inhibitor responses. Many mutations in the exon region of somatic cells lead to an increase in the production of neoantigens recognized by T cells, thereby enhancing the antitumor immune response. As a result, patients with high TMB may develop a stronger immune response and be more sensitive to immune checkpoint inhibitor therapy [28]. For example, activation of the Keap1/Nrf2/HO-1 pathway and ferritin phage-mediated ferroptosis contributed to EMT inhibition of GC cell proliferations and altered the cellular redox environment [29]. Besides, ferroptosis-related lncRNA regulated the invasiveness of GC. lncRNA-BDNF-AS/WDR5/FBXW7 axis regulated VDAC3 ubiquitination and then mediates ferroptosis in GC peritoneal metastasis [30]. Ferroptosis can also inhibit drug resistance in GC. Ferroptosis induced by ATF3 overexpression can reduce cisplatin resistance in GC [31]. Other studies have shown that change in lipid metabolism around cancer cells under stress determines the ferroptosis sensitivity of GC [32]. Interestingly, TMB was significant in high-risk group. Therefore, we can reasonably speculate that FRGs were key genes for the prognosis of GC.

In this study, through TCGA dataset, we identified two FRGs subclusters (C1 and C2) through NMF cluster analysis. There were significant differences in the survival status of the two clusters. WGCNA was applied to identify ferroptosis subcluster-related modular genes. Through Cox regression analysis, we selected the prognostic genes related to ferroptosis and established the prognostic risk-scoring model. We also used GSE84437 dataset to externally verify the prognostic risk-scoring model. At the same time, we integrated clinicopathological features and risk-scoring model to construct a nomogram for clinical application. The function of TIME and TMB was studied to preliminarily clarify the mechanism of its influence on prognosis, so as to provide basis for clinical diagnosis, individualized immune-targeted therapy, and antitumor drug resistance.

Based on the above intersection genes, a novel prognostic model integrating 8 FRGs (CTHRC1, PDPN, PLOD2, GFPT2, ABCA1, GPR176, SERPINE1, and DUSP1) was firstly constructed. For example, CTHRC1 was used as a marker of colorectal cancer (CRC) intratumoral metastasis, and Zhang et al. confirmed that CTHRC1 promoted liver metastasis of CRC and earlier predicted targets by TGF-*β* remodeling infiltrating macrophage signaling [33]. PDPN (+) CAF, the representative of immunosuppressive microenvironment of lung adenocarcinoma, can induce macrophage M2 polarization and inhibit immune-related lymphocytes, serving as a bridge between fiber microenvironment and immunosuppression [34]. In patients with large tumor or solid tumor metastases, PDPN expression induced poor prognosis in cancer-associated fibrous tissue [35]. And PLOD family genes can affect the progression and prognosis of human digestive tract tumors. As a member of them, PLOD2 was not only related to the histological grading of pancreatic cancer, but also overexpressed in TP53 and KRAS types [36]. GFPT2 in the study was also closely related to the prognosis, microenvironment, immunity, and drug sensitivity of other digestive system tumors, and the specific internal mechanism remained to be further studied [37]. Besides, GFPT2 expression was inhibited by the oxidative stress regulator GSK3-*β*. GFPT2 was a marker of poor prognosis in the D492 EMT model of breast cancer, which controlled growth and invasion [38]. Compared with other FRGs, ABCA1 may be a tumor suppressor that was methylated after dysregulation of transforming growth factor-*β* signaling in ovarian cancer, presenting a poor prognosis. In contrast, SERPINE1-upregulated GC patients showed poor OS and PFS. It was considered that it may regulate VEFF and JAK-STAT3 inflammatory signaling pathways to affect GC cell proliferation and migration [39]. DUSP1 was also observed to be an oncogene associated with drug resistance during cancer intervention. At present, the role of GPR176 in GC prognosis is unclear. Upregulation of GPR176 stimulates the function of Sirtuin6. Sirtuin6 overexpression inhibited breast cancer stem cell biogenesis in cells with a PI3K mutation and murine PyMT mammary tumor progression in vivo [40]. And there are few reports on the use of ferroptosis to correlate TIME and TMB in GC prognostic models.

FRGs plays a crucial role in TME, as shown in Figure 10(a). By comparing the immune infiltration between risk score groups, we found that T cells were the most extensively infiltrated immune cells in GC samples, and macrophages, mast, and T helper cells showed significantly different distribution [41, 42]. Some studies have reported the immune potential of tertiary lymphocyte structures around primary GC, in which DC was a set that affected the reactivity, cytotoxicity, and monitoring escape status of anticancer cells [43]. Clinical validation of GC suggested that TAM M1 macrophages were associated with antitumor activity. M2 promoted pro-angiogenic and immunosuppressive signals in tumors, such as diffuse GC subtypes [44]. In the high-risk group, T cell infiltration levels were elevated. It meant that high-risk group with FRGs had a better chance of taking advantage of cellular immune-personalized therapy regiments. We also enriched biological signaling pathways, for instance, PI3K−Akt, TGF-*β*, and JAK-STAT. Recent studies have indicated that MAPK pathway participated in resistance to GC ferroptosis. And inhibition of MAPK signaling can protect GC cells from ferroptosis [45]. In addition, activated TGF-*β* was identified to promote ferroptosis [46]. In our study, the tumor purity of TME decreased, and the infiltration of stromal and immune cells increased significantly. Low purity of GC in high-risk group was associated with poor prognosis. Therefore, these results detailed the conditions and ways in which FRGs regulate GC development, which may be conducive to further study of immune escape surveillance. In addition, ferroptosis-related reactive oxygen species and iron uptake could lead to somatic nonsynonymous mutations and microsatellite instability, resulting in increasing immunogenicity and immune infiltrates [47, 48], which was consistent with our findings.

There are still a few limitations. First, all data sources came from public databases. There is a lack of real world samples and prospective clinical data validation. Secondly, ferroptosis is not a unique mechanism in GC, and whether ferroptosis is involved in the mechanism of TIME is still uncertain. Besides, FRGs obtained from previous studies may be incomplete, which requires further improvement of the FRGs database from future studies. Finally, whether prognosis FRGs directly regulates the ferroptosis process in GC requires further experimental verification.

## 5. Conclusion

In conclusion, the risk-scoring model based on 8 ferroptosis subcluster-related modular genes has shown outstanding advantages in predicting patient prognosis. The interaction of ferroptosis in GC development may provide new insights into exploring molecular mechanisms and targeted therapies for GC patients.

## Data Availability

Data available on request from the authors.
